# Influence of Speciation of Thorium on Toxic Effects to Green Algae *Chlorella pyrenoidosa*

**DOI:** 10.3390/ijms18040795

**Published:** 2017-04-10

**Authors:** Can Peng, Yuhui Ma, Yayun Ding, Xiao He, Peng Zhang, Tu Lan, Dongqi Wang, Zhaohui Zhang, Zhiyong Zhang

**Affiliations:** 1 School of Public Health, University of South China, Hengyang 421001, China; vintagecancan@icloud.com; 2 Key Laboratory for Biomedical Effects of Nanomaterials and Nanosafety, Institute of High Energy Physics, Chinese Academy of Sciences, Beijing 100049, China; dingyy@ihep.ac.cn (Y.D.); hx421@ihep.ac.cn (X.H.); pengzhang@ihep.ac.cn (P.Z.); lantu@ihep.ac.cn (T.L.); dwang@ihep.ac.cn (D.W.); 3 School of Physical Sciences, University of the Chinese Academy of Sciences, Beijing 100049, China

**Keywords:** thorium, speciation, toxicity, green algae, *Chlorella pyrenoidosa*

## Abstract

Thorium (Th) is a natural radioactive element present in the environment and has the potential to be used as a nuclear fuel. Relatively little is known about the influence and toxicity of Th in the environment. In the present study, the toxicity of Th to the green algae *Chlorella pyrenoidosa* (*C. pyrenoidosa*) was evaluated by algal growth inhibition, biochemical assays and morphologic observations. In the cultural medium (OECD TG 201), Th(NO_3_)_4_ was transformed to amorphous precipitation of Th(OH)_4_ due to hydrolysis. Th was toxic to *C. pyrenoidosa*, with a 96 h half maximum effective concentration (EC_50_) of 10.4 μM. Scanning electron microscopy shows that Th-containing aggregates were attached onto the surface of the algal cells, and transmission electron microscopy indicates the internalization of nano-sized Th precipitates and ultrastructural alterations of the algal cells. The heteroagglomeration between Th(OH)_4_ precipitation and alga cells and enhanced oxidative stress might play important roles in the toxicity of Th. To our knowledge, this is the first report of the toxicity of Th to algae with its chemical species in the exposure medium. This finding provides useful information on understanding the fate and toxicity of Th in the aquatic environment.

## 1. Introduction

Thorium (Th) is an actinide that occurs naturally as ^232^Th with a very long half-life of 1.4 × 10^10^ years. In the environment, Th exists predominantly in the tetravalent state, and is a trace constituent in phosphates, simple and multiple oxides, and silicates [1,2]. Th is used for making ceramics, welding rods, camera and telescope lenses, fire brick, heat resistant paint, and metals used in the aerospace industry [3]. In recent years, Th has attracted more and more attention because it has the potential to be used as a cleaner, safer, and more abundant nuclear fuel.

People will always be exposed to small amounts of Th through inhalation, ingestion and skin penetration, because Th is naturally ubiquitous in air, water, soil and biological materials. Individuals that work in the mining, milling or thorium industries may be exposed to more Th than usual. There is evidence that breathing in Th dust increases the risk of lung and pancreatic cancer. Exposure to Th also causes bone cancer because Th can be stored in bone [4]. In the 1930s and 1940s, ThO_2_ was used as a radiographic contrast agent (thorotrast). After intravascular injection, ThO_2_ was found mainly in reticuloendothelial systems such as liver, spleen and bone and has been associated with an increased incidence of liver disease [5]. Recently, studies on the toxicity of Th at cellular and molecular levels have also been carried out. Oliveira et al. [6] found that Th was not toxic to human lymphocytes at concentration range from 0 to 1 mM. Allred et al. [7] reported that an iron-binding protein called siderocalin could bind and transport actinides such as Th, Pu, Am, etc., into cells. It was considered to be a major advance in understanding the biological chemistry of radioactive actinides.

The aquatic environment is the final sink of all pollutants, but there are currently only a few published articles on aquatic toxicity of Th. Researchers from Russia investigated the effect of ^232^Th to the fresh water green alga *Chlorella pyrenoidosa* and found that the 24 h EC_50_ value of Th was 15.4 μM and the presence of caffeine significantly increased Th toxicity [8]. However, de Queiroz et al. [9] reported that the growth of two green microalgae *Monoraphidium* sp. and *Scenedesmus* sp. was resistant to Th. Adverse effects on cell growth were observed only for concentrations higher than 215 μM. The authors postulated that the presence of the NO_3_^−^ in the stock solution of Th could have been used by the cells as a nutrient source and EDTA presented in ASM-1 medium acted as a complexing agent for Th, preventing its interaction with the microalgae. The effects that Th has on aquatic invertebrate and vertebrate species have also been studied. Borgmann et al. [10] evaluated the toxicity of 63 metals and metalloids including Th to freshwater amphipod *Hyalella azteca.* Lethal concentration resulting in 50% mortality (LC_50_) of Th was correlated with the hardness of the cultural media. The LC_50_s of Th in tap water was higher than that in soft water. Correa et al. [11] tested the effect of 15 days of waterborne Th exposure on accumulation, metabolic and oxidative parameters in the bile, gills, liver, muscle, brain, skin, kidney and blood of the silver catfish (*Rhamdia quelen*). The concentrations of Th in gills and skin were the highest among all the organs. CAT and GST activities in the liver and muscle were altered by Th treatment. A prolonged exposure of 30 days to Th obtained similar results [12]. It is well known that in the aquatic environment, the most important factor that influences the toxicity of heavy metals is their chemical species. Ionic Th tend to form hydrolyzed species and/or insoluble residues in aqueous solutions at pH higher than 3. In most reported studies, the aquatic organisms were treated with Th(NO_3_)_4_, but the transformation of Th in the exposure media (modified reconstituted water) was almost never analyzed. Recently, our group demonstrated that composition of exposed media significantly influences the Th species and Th was present as particulate ThO_2_ in the exposure medium of *D. magna*. The 24 h and 48 h EC_50_ of ThO_2_ were 7.3 and 4.7 μM, respectively [13]. This suggests that more attention should be paid to the toxicity of Th species that are present in insoluble forms.

Algae, primary producers in the aquatic system, are ubiquitous and have colonized almost every part of the world. Using green algae (e.g., *Scenedesmus obliquus*, *Chlorella pyrenoidosa*, etc.) to investigate the aquatic toxicology of chemicals is advantageous, since they are easy to culture and sensitive to pollutants. The objective of this study was to access the toxicity of Th to *C. pyrenoidosa* on the basis of the chemical speciation of Th in the exposure medium. The EC_50_, chlorophyll a concentrations and ROS levels of *C. pyrenoidosa* treated by Th were determined. The morphological changes of algal cells and uptake of Th were observed by SEM and TEM. This work will provide understanding on the toxicity of Th to the aquatic environment.

## 2. Results and Discussion

### 2.1. Chemical Species of Th in the Media

**Figure 1:**
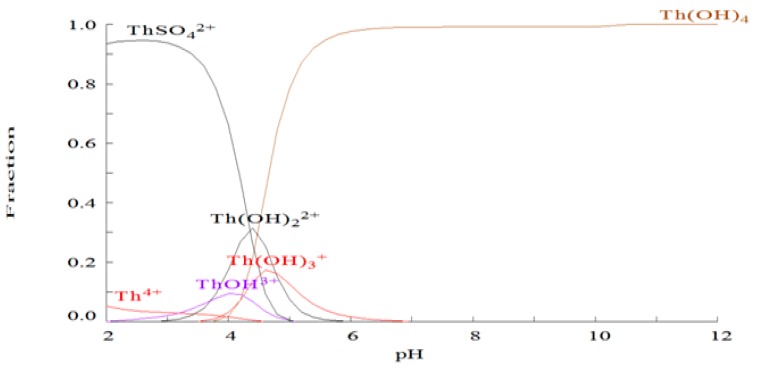
Distribution diagram of different forms of Th at the highest treated concentration in dependence on pH values.

Th^4+^ ions tend to form hydrolyzed species and/or insoluble residues in aqueous solutions [14]. Moreover, the formation of colloidal species of this element is known to start at very low pH. Considering the complexity of OECD medium, the MEDUSA program was used to calculate the distribution diagrams of different chemical forms of Th in the exposure media. As shown in Figure 1, the hydroxo-complexes (Th(OH)_2_^2+^, Th(OH)^3+^ and ThOH^3+^) aggrandized with the increase of pH values; as a consequence, the concentration of free Th^4+^ decreased. Since the pH values of Th-containing solutions were all above 7.0, according to the distribution diagrams there were no free Th^4+^ ions in the exposure media, with all of Th being present as Th(OH)_4_. It has been reported that the Ksp value for Th(OH)_4_ was only 5.34 × 10^−33.6^ [15], which means the solubility of Th(OH)_4_ in the water was extremely low.

### 2.2. Effects of Th on Algal Growth

**Figure 2:**
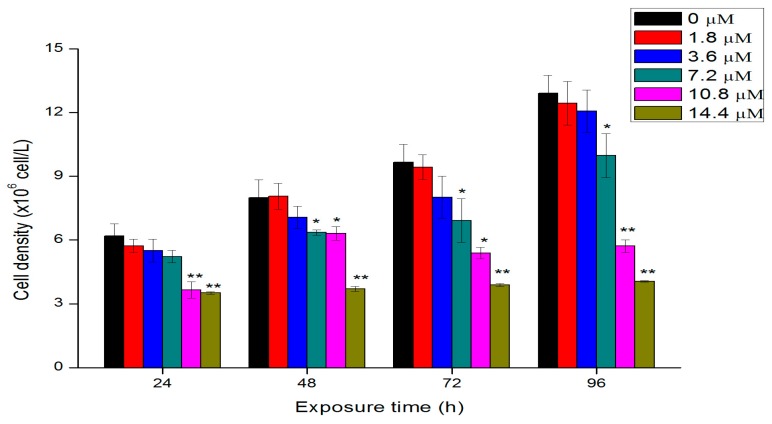
Cell density of algae cells after exposure to different concentration of Th. Data are showed as mean value ± standard deviations (SD). Significant difference of the experimental value compared with each control was marked with “*” (*p* < 0.05) or “**” (*p* < 0.01).

The concentration- and time-dependent effects of Th to *C. pyrenoidosa* are shown in Figure 2. At concentrations of less than 7.2 μM, Th had no effect on algal growth, while it showed significant toxicity at higher concentrations at each exposure time. The 24-, 48-, and 72-, and 96-h EC_50_ values of Th to *C. pyrenoidosa* are calculated and summarized in Table 1. The growth inhibition increased with the increasing of exposure time. This result was consistent with other previous reports, in which the toxicity of lead [16], cadmium [17] and chlorine [18] to algae was related to the incubation time. After 96 h exposure, the inhibition rates relative to the control group of 7.2, 10.8, and 14.4 μM Th were 22.5%, 55.1%, and 67.8%, respectively. The 24 and 96 h EC_50_ values were respectively 18.5 and 10.4 μM, which was comparable to the results of Evseeva et al. [8].

### 2.3. Effects of Th on Chlorophyll a Contents

**Figure 3:**
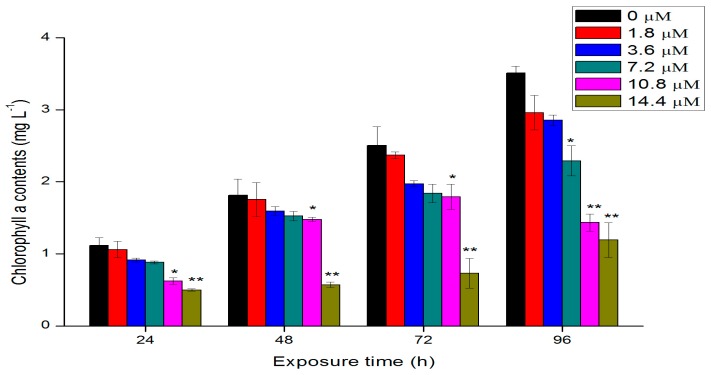
Chlorophyll a contents of algae cells after exposure to different concentration of Th. Data are showed as mean value ± standard deviations (SD). Significant difference of the experimental value compared with each control was marked with “*” (*p* < 0.05) or “**” (*p* < 0.01).

Chlorophyll, one kind of photosynthetic pigment, is the basis for photosynthesis of algae cells. The growth status of cells can be reflected by monitoring the intracellular contents of chlorophyll. The chlorophyll a contents of algal cells after being treated with different concentrations of Th are shown in Figure 3. The general trend of chlorophyll changes was similar to that of the growth (Figure 2). With the increase of exposure time and concentrations, the contents of chlorophyll a of algae cells decreased gradually. After 96 h exposure, the content of chlorophyll a in the cells exposed to 10.8 and 14.4 μM Th was reduced by 34.5% and 58.6% respectively compared with that of the control group (*p* < 0.01). The reduction of chlorophyll contents after exposure to Th indicated the ability of the cells to synthesize chlorophyll or the photosynthetic reaction center complexes was impacted. Similar to effects of other heavy metals, such as lead and cadmium, on the green algae [17], the decreased chlorophyll content may result in a decrease in the photosynthetic activity and chlorosis and indicates that the ability of the cells to synthesize chlorophyll or the photosynthetic reaction center complexes was impacted after exposure to Th.

### 2.4. Morphological Changes

**Figure 4:**
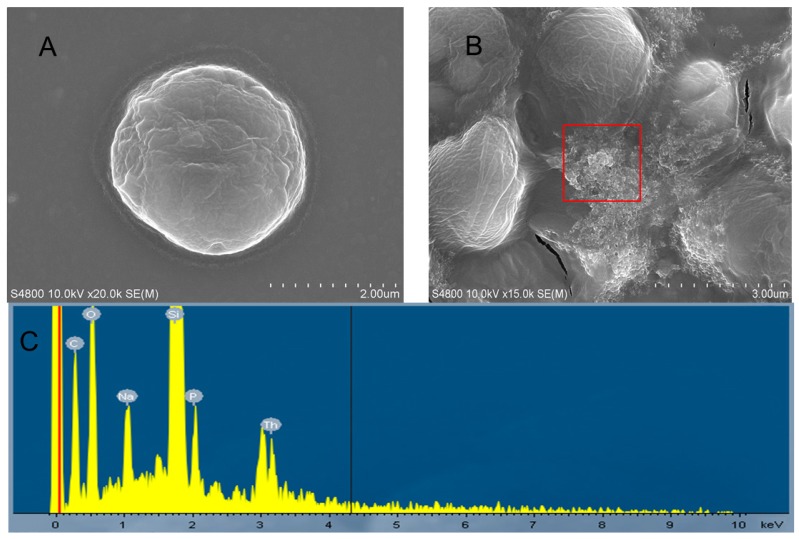
SEM images of *C. pyrenoidosa* after exposure for 96 h. (**A**) control algal cell; (**B**) algal cells treated with 14.4 μM Th; (**C**) energy dispersive spectroscopy (EDS) analysis of the area marked by the red box in panel **B**.

The SEM images in Figure 4 show morphology differences between the control and exposed cells. As shown in Figure 4A, a typical untreated cell was intact and enclosed with a rigid cell wall. After being incubated with 14.4 μM of Th for 96 h, however, the algae cells were aggregated together and the cells were shrunk and distorted (Figure 4B). Moreover, a number of particle aggregates were attached on the surface of the treated cells, indicating the strong agglomeration between particles and cells. The EDS spectrum of the red box marked in Figure 4B shows the presence of Th (Figure 4C), which suggests the attachment of Th on the external surface of algae cells. As mentioned above, Th was transformed into Th(OH)_4_ in the medium. Therefore, the observed precipitation adsorbed to the surface of alga cells was probably Th(OH)_4_, which will increase the opportunity contacting with algal cells, and may thus contribute to the algal toxicity of Th. It has been reported that heteroagglomeration may lead to direct and indirect toxicity to algae through internalization, physical damage, oxidative stress, and/or shading effects [19,20]. In this study, the heteroagglomeration between the precipitation of Th(OH)_4_ and algae cells could possibly lead to a reduction in the light available to cells and thus decrease the chlorophyll contents. On the other hand, it might be a mechanism of egodefence that the exposed cells aggregated together to decrease the physical contact with Th, as reported by Zhao et al. [21], who showed that the aggregated algae cells act as a barrier to prevent the direct damage of CuO NPs to the cell wall and membrane.

### 2.5. Ultrastructural Alterations

**Figure 5:**
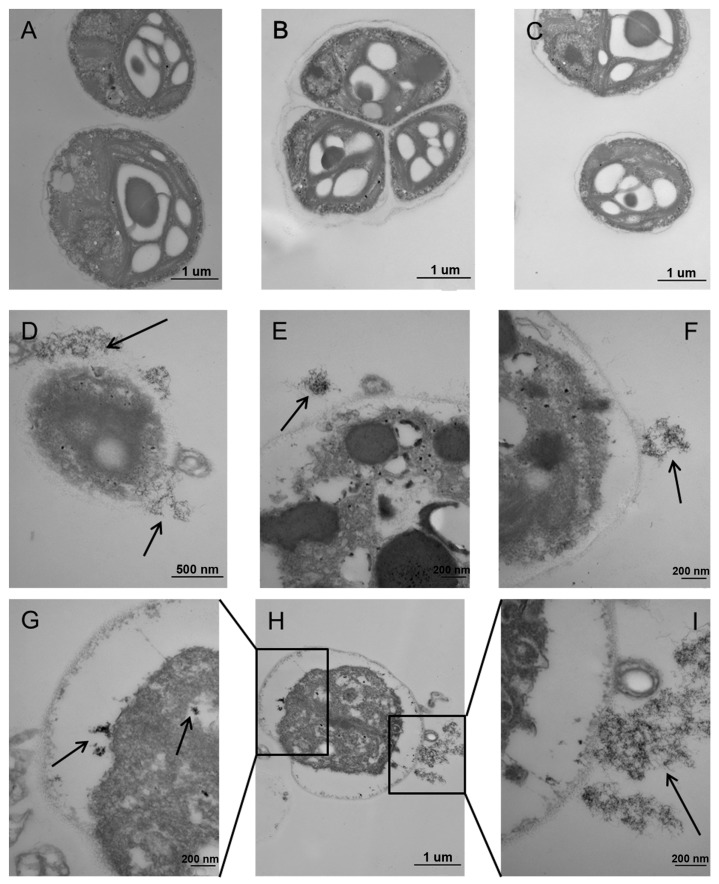
Transmission electron microscopy (TEM) images of ultrathin slices of C. *pyrenoidosa* after exposure to Th for 96 h. (**A**–**C**) control algae cells; (**D**–**I**) algae cells treated with 14.4 μM of Th. (**G**,**I**) are higher magnification of the areas marked by the black boxes in (**H**). The black arrows denoted the precipitation of Th.

TEM images in Figure 5 show the ultrastructural differences of algae cells between the control and the exposed group. In the control group, all the cells had a well-shaped structure. A clear nucleus was observed and the cytoplasm was closely attached to the cell membrane (Figure 5A–C). In contrast, the algal cells exposed to 14.4 μM Th were deformed with serious plasmolysis (Figure 5E,F,H), indicating the great algal toxicity of Th. In particular, as shown in Figure 5E, the cytoplasm obviously shrunk and the cell nucleus was blurry compared with the untreated cells, which may lead to a dysfunction of chloroplasts, such as the limitation of nutrient uptake by algae cells [22]. The cell wall/membrane of the exposed alga cells were irregular and were attached with a number of amorphous precipitations (Figure 5E,F,I, black arrows), and this might result in cell wall and membrane damage as shown in Figure 5D. Moreover, Th compounds could also be found inside the cells (Figure 5G, black arrows). As shown by SEM and TEM, Th in the form of Th(OH)_4_ was nano-sized and internalized by alga cells. These nano-sized Th(OH)_4_ deposits may impair normal cellular processes.

### 2.6. Oxidative Stress

**Figure 6:**
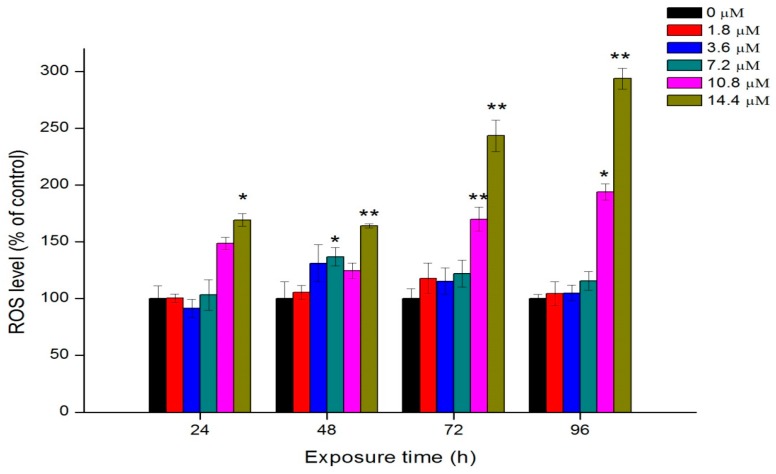
ROS generation of algae cells after exposure to different concentration of Th. Data are showed as mean value ± standard deviations (SD). Significant difference of the experimental value compared with each control was marked with “*” (*p* < 0.05) or “**” (*p* < 0.01).

Exposure of algae cells to Th induced an increase in intracellular ROS levels (Figure 6). At the first 24 h, the difference of ROS between the control group and 1.8, 3.6, 7.2 and 10.8 μM of Th was insignificant, while the ROS level was significantly increased by 169.2% at 14.4 μM of Th compared to the control group (*p* < 0.05). At 48 h and 72 h, no clear does-response relationship was found, except that the ROS formation significantly increased at 14.4 μM of Th (*p* < 0.01). At 96 h, the ROS levels were enhanced approximately 2 and 3 times at exposure of 10.8 and 14.4 M Th, respectively. Elevated intracellular ROS levels may result in oxidative damage to DNA and other macromolecules and this may subsequently lead to cell death.

The mechanism of the toxicity of Th to aquatic organisms is still unknown. Common heavy metal pollutants (e.g., Cd, Pb, Hg, etc.) show high affinity for thiol containing biomolecules (GSH and sulfhydryl proteins) and act as a catalyst in Fenton-type reactions, producing oxidative damage [23]. Since Th was present as an insoluble Th(OH)_4_ in the exposure medium, the biological behavior of Th was probably different from those above mentioned heavy metals. The TEM images show that nano-sized Th(OH)_4_ was deposited both inside and outside the cells. Recent progresses in the aquatic toxicity of engineered nanomaterials suggest that insoluble nano-sized particles might exhibit toxicity to green algae by the following processes: (1) the shading effect may attenuate the photosynthesis by reducing light transmittance; (2) the heteroagglomeration and physical interaction may lead to the internalization of nanoparticles, cell membrane disruption and endocyte outflow; (3) the permeation and entry of nanoparticles into the cells may induce the elevation of intracellular ROS levels and the membrane lipid peroxidation [19,20,21,24,25,26]. All these processes might contribute to the toxic effects of Th to *C. pyrenoidosa*. However, understanding the underlying mechanism requires further investigations.

## 3. Materials and Methods

### 3.1. Materials and Chlorella pyrenoidosa Culture

Th was used in the form of Th(NO_3_)_4_∙5H_2_O with purity over 99%. All chemicals were analytical grade and were purchased from Beijing Chemical Plant. Freshwater algae *Chlorella pyrenoidosa* (*C. pyrenoidosa*) was obtained from the Institute of Hydrobiology, Chinese Academy of science, Wuhan, China. The algae were cultured in 100 mL conical flasks containing complete Organization for Economic Co-operation and Development (OECD) 201 medium [27]. The flasks were placed on a shaker (95 ± 5 r/min) in the bed temperature incubator. The temperature of the incubator was set at 24 ± 1 °C under illumination of 3000 ± 10% l× light intensity, with a 12-h light and 12-h night daily cycle. During the exponential growth phase, the algae cells were calculated by a hematocytometer and the cell density was monitored at 680 nm (OD_680_) with a microplate reader (infinite M200 PRO). According to the results of hematocytometer and OD_680_ values, there was a correlation between the cell density of *C. pyrenoidosa* and the optical density (OD_680_) values. The regression equation was calculated as *y* (×10^6^ mL^−1^) = 50.97 OD_680_ − 0.15 (*R*^2^ = 0.99).

### 3.2. Algal Growth Assays

Following the OECD 201 algal growth-inhibition test guidelines, algae cells in the logarithmic growth phase were used for all experiments. The cells were collected, washed, and diluted to the initial concentration of 2 × 10^6^ cells L^−1^ for the exposure of Th. Th(NO_3_)_4_ 5H_2_O were added to the axenic algae 201 medium to reach the final Th concentrations at 0, 1.8, 3.6, 7.2, 10.8, and 14.4 μM. After the addition of Th, the pH values of the treated algal media were all around 7.4. The algae cells in the control group without Th were conducted following the same procedure. All treatments were performed in triplicate. The growth of algae cells was examined by monitoring the OD_680_ values after different exposure time (1, 2, 3 and 4 days). Percent inhibition of growth was calculated at each time for the estimation of EC_50_ and 95% confidence interval using Probit analysis in SPSS software (version 18, IBM, Armonk, NY, USA).

### 3.3. Chlorophyll a Fluorescence Measurements

The fluorescence intensity of chlorophyll a was measured at different exposure times, following a procedure described by Jeffrey and Humphrey [28]. Four milliliters of ethanol was added to 1 mL algae suspension of each group to extract the chlorophyll. After 3 h reaction in the dark, the mixing suspension was measured by a fluorescence spectrophotometer (RF-5301PC). The excitation and emission wavelengths were 420 and 671 nm, respectively. According to the result of preliminary experiment, concentrations of chlorophyll a and the fluorescence intensity have a dose relationship (*I*_F_ = 179.50*c* + 5.62, *R*^2^ = 0.98).

### 3.4. Th Speciation in the Cultured Medium

To determine the actual chemical species of Th present in the cultural medium, make equilibrium diagrams using sophisticated algorithms (MEDUSA program) was used for the construction of distribution diagrams. The basic parameters, including equilibrium constants that are needed for the calculation of equilibrium diagrams were in the program database. The program was written by Ignasi Puigdomenech from the Inorganic Chemistry department of Royal Institute of Technology, Stockholm, Sweden [29]. The MEDUSA program is a freeware and is available at http://www.kemi.kth.se/medusa.

### 3.5. SEM And TEM Observations

After being exposed to 0 or 14.4 M of Th for 96 h, the algae cells were collected by centrifugation (400× *g*) for 5 min at 4 °C. Then the supernatant was removed, the collected algae cells were washed with phosphate-buffed saline (PBS, pH 7.4) for three times. The washed cells were then resuspended with PBS at certain concentration and placed on sample holder for observation. The morphology of algae was observed with a scanning electron microscope (SEM, S-4800, Hitachi, Tokyo, Japan), equipped with the energy dispersive spectroscopy (EDS, EMAX-250, HORIBA, Kyoto, Japan).

The transmission electron microscopy (TEM) observation of algae cells followed a modified procedure described by Xia et al. [30]. All samples (treated and untreated cells) were postfixed in 1% osmic acid for 1 h and washed with phosphate butter solution (PBS, pH 7.0) for 3 times. Dehydration process of the samples was conducted in increasing concentrations of acetone at room temperature. The samples were permeated and impregnated in resin for 5 h. Ultrathin sections were made and placed on Cu grids for imaging. The ultrastructure of algae cells were observed with TEM (JEM-1230, JEOL, Tokyo, Japan).

### 3.6. Oxidative Stress

ROS generation of algae cells under different treatments was detected using 2,7-dichlorodihydrofluorescein diacetate (DCFH-DA), which is an oxidation-sensitive fluorescent probe dye. The ROS was measured according to the approach described by Saison et al. [31]. After exposure to Th for 0–96 h, 20 μL of DCFH-DA (100 μM) was added to 180 μL of algae suspension to reach the final concentration at 10 μM. Then the algal cells were washed three times to remove the unbound DCFH-DA. DCFH-DA could be transformed into H_2_DCF by intracellular esterase if they enter cells. When intracellular ROS was generated, intracellular H_2_DCF could be deacetylated and then oxidized to the highly fluorescent dichlorofluorescein (DCF). The fluorescence intensity of DCF, which indicated the extent of ROS generation, was measured using a microplate reader (infinite M200 PRO). The excitation and emission wavelengths were 488 and 525 nm, respectively. The ROS levels in the treated groups were expressed as percentages relative to the control group.

### 3.7. Statistical Analysis

Data are reported as mean ± S.D. The significant differences between the control and the treatments were analyzed by one-way ANOVA with least significant difference (LSD) test or Kruskal-Wallis H ANOVA with Mann–Whitney *U* test. Analysis was performed using the IBM SPSS (version 18, IBM). The significant level was set at *p* < 0.05 (*) or *p* < 0.01 (**).

## 4. Conclusions

This study investigated the toxicity of Th to *C. pyrenoidosa* on the basis of its chemical species in the cultural medium. Th in the form of Th(OH)_4_ could inhibit the growth of algae cells, reduce chlorophyll contents, and enhance the intracellular levels of ROS. Th-containing precipitation was attached on the surface of algal cells as determined by SEM observation combined with EDS. TEM images showed the plasmolysis, membrane damage, and ultrastructural changes of the exposed algal cells. Overall, the direct physical interaction of agglomerations with algal cells and generation of intracellular ROS were the main reasons for the toxicity of Th to *C. pyrenoidosa*. This study significantly advances our understanding of the potential toxicity of Th to aquatic species. In future studies, more attention should be paid to the toxicity of Th in insoluble forms such as Th(OH)_4_.

## Figures and Tables

**Table 1:** The EC_50_ values with 95% confidence interval (CI) of Th to *C. pyrenoidosa* at different exposure time.

Time (h)	EC_50_ (μM)	95% CI (μM)
24	18.5	10.9 26.7
48	16.7	14.7 21.7
72	11.8	10.2 14.1
96	10.4	7.9 15.9
